# Implementing pharmaceutical track-and-trace systems: a realist review

**DOI:** 10.1136/bmjgh-2020-003755

**Published:** 2021-05-28

**Authors:** Joeke Kootstra, Tineke Kleinhout-Vliek

**Affiliations:** Erasmus School of Health Policy & Management, Erasmus University Rotterdam, Rotterdam, The Netherlands

**Keywords:** health policy, health systems, public health, review

## Abstract

**Introduction:**

One way to prevent falsified medical products from entering the regulated pharmaceutical supply chain is to implement a pharmaceutical track-and-trace system (PTTS). Such systems in the most extensive versions generally mandate a scan at every point of contact with the medical product: from the point of entry to dispensation. There have been several attempts to implement such systems; for example, a ‘full’ PTTS in Turkey and the more pared-down version offered by the European Union’s Falsified Medicines Directive and Delegated Act. This study aims to identify facilitators and barriers to implementing (elements of) a PTTS, with the Turkish system used as a benchmark.

**Methods:**

We conducted a ‘realist’ review, which synthesises literature and aims to establish how a specific technology works, for whom, under which circumstances. We searched Embase, Medline Ovid, Web of Science, Cochrane Central and Google Scholar databases, yielding 2,790 scholarly articles. We selected 21 for review.

**Results:**

Implementation of PTTS elements has been attempted in different compositions in several primarily high-income and middle-income countries. Factors that affected implementation included stakeholders like the government and supply chain actors, the coordination between them, and their awareness, knowledge, and skills, as well as regulation and legislation, monetary investments, and technical and digital requirements.

**Conclusion:**

The interplay between contextual factors is crucial for successful PTTS implementation. Specifically, the findings indicate that aligning the incentives for all actors and allowing for adjustments in a continuous implementation process will likely facilitate implementation.

**Key questions**

**What is already known?**

- Substandard and falsified medicines threaten patient safety and health systems generally, and these are present everywhere.
- One way to hinder falsified medicines from entering a supply chain is to implement a pharmaceutical track-and-trace system.
- Several countries have implemented or attempted to implement pharmaceutical track-and-trace systems; Turkey has implemented a complete and functioning system.

**What are the new findings?**

- Political, economic and social contextual factors (government support, supply chain actors and the coordination between them, awareness, knowledge, and skill of supply chain actors, regulation and legislation, financial investments, and technical and digital requirements) affect the implementation of pharmaceutical track-and-trace systems.
- The interplay between these contextual factors strongly affects implementation.

**What do the new findings imply?**

- Aligning the incentives for all actors and allowing for adjustments during implementation will facilitate the implementation of pharmaceutical track-and-trace systems.
- Further research could address the interplay between contextual factors and how these can be aligned to facilitate implementation.

## Introduction

Poor-quality medical products threaten patients through toxicity, increased antimicrobial resistance, and undermining of health systems.^1^ As such, they create barriers on the road to achieving Universal Health Coverage.^2^ In 2017, the WHO’s Global Surveillance and Monitoring System for substandard and falsified medical products analysed 1,500 cases of suspect medicines, concluding that the problem exists worldwide.^3^ Recently, steps have been taken concerning alignment on terminology on substandard and falsified medical products (SFMPs), with substandard defined by the WHO as ‘authorised medical products that fail to meet either their quality standards or specifications or both’ and falsified defined as ‘medical products that deliberately or fraudulently misrepresent their identity, composition or source’.^4^ The Oxford Statement and Medicines We Can Trust Campaign followed soon after.^5^

Pharmaceutical track-and-trace systems (PTTSs) may offer a (partial) solution to the problem of falsified medical products in particular.^6^ PTTSs work by providing medicine packages with a unique code, like a barcode or a radio-frequency identification code (RFID, which may transmit digital data without necessarily being within the reader’s line of sight).^7^ Scanning the code authenticates the medical product. A central management system stores the information retrieved on scanning.^8^ Scanning provides information from the central system, like expiry dates, and information concerning recalls or falsification alerts.^6^ Several forms of PTTS exist. A ‘full’ PTTS, as implemented in Turkey in 2012,^9–11^ can follow all medical products throughout legal supply chains from the point of entry to dispensation through barcode verification at every transfer of ownership. Not all PTTSs require a scan at every point; ‘end-to-end’ systems require commissioning medicine packages at production and decommissioning at the point of supply to the final user.^12^ Such pared-down forms of PTTSs exist in countries such as India,^13^ Argentina, Brazil and China,^14^ as well as in the European Union (EU) through its Falsified Medicines Directive and Delegated Act.^15^ A PTTS may aid in preventing falsified medical products reaching patients in three primary ways. First, it may improve identification of falsified, recalled or expired medicines.^16^ Second, it can facilitate the recall of (partial) batches in case of irregularities.^12^ Third, a PTTS may improve medical products’ quality by preventing these medical products from entering the market.^7^

Implementation of a PTTS is not straightforward. One known factor that may hinder PTTS implementation is limited pharmaceutical regulatory capacity.^17^ Such ‘contextual’ factors influence the implementation of extensive technical systems.^18^ Implementation in a ‘real-world’ context requires effort.^19^ Thus, contextual factors significantly impact the implementation of PTTSs, but few papers address their interaction during implementation.^20 21^ In this paper, we survey the peer-reviewed literature on (attempted) implementation of PTTSs, focusing our efforts on the contextual factors described to have affected these (attempted) implementations. We answer the following research question: what political, economic, and social contextual factors facilitate or hinder the implementation of PTTSs, and what are the implications for future PTTS implementations?

## Methods

We applied the realist review method to synthesise peer-reviewed literature on contextual factors that may influence the implementation of a PTTS according to the RAMESES publication standard.^22^ A realist review seeks to survey the literature (which may be peer-reviewed, grey or both) on a specific technology to understand how this technology may work, for whom, in what context. Employing a realist review, we identified the mechanisms (that is, elements of a PTTS) and contexts (that is, political, economic and social contextual factors) that have led to various outcomes described in the literature. By retrieving such context–mechanism–outcome combinations and, based thereon, formulating programme theories, a deeper understanding of the intervention and how it could potentially work can be reached.^23^ Especially for complex healthcare interventions, realist reviews may uncover the ‘black box’ holding information on the outworking of an intervention-in-context.^24^

The realist review method prescribes explicating the theoretical underpinnings of how a particular mechanism ought to work in the form of programme theories.^23^ For this paper, we have built on two explicit programme theories. The first holds that PTTSs may help prevent falsified medical products reaching patients, as described in the introduction. This programme theory has influenced the data collection specifically, with Turkey’s Ilaç Takip Sistemi (ITS, Turkish for ‘PTTS’) taken as a benchmark case. We did so for three reasons. ITS was the first to be adopted in 2012; second, it is a relatively extensive, ‘full’ PTTS with many different elements^25^; and third, ITS is considered relatively successful.^9–11^ It is said to have prevented sales of both smuggled and counterfeited drugs as well as barcode scams.^25^ We derived the list of mechanisms described in this paper directly from ITS. The second programme theory holds that PTTSs are innovations-in-context, and contextual factors (‘contexts’) will impact the implementation process specifically (see the Introduction section). Aggregating these past implementations, we hold, will give insight into the contextual factors that might impact future PTTS implementation processes. It will also help refine these programme theories concerning the implementation of PTTSs, and in this way, ‘enable decision-makers to reach a deeper understanding of the intervention and how it can be made to work most effectively’.^23^

We conducted a systematic literature search in the Embase, Medline Ovid, Web of Science, Cochrane Central, and Google Scholar databases. The search terms (see box 1) comprised of two main parts connected by ‘AND’; first the track-and-trace part, searching for bar codes, RFID, track-and-trace or end-to-end. The second part focused on drugs and pharmaceuticals, and pharmacies and other physical locations.

Box 1 **Search terms used in Embase**

(‘bar code’/de OR ‘barcode scanning’/de OR ‘radiofrequency identification’/exp OR (((track*) NEAR/3 (trace OR traced)) OR bar-cod* OR barcod* OR serialization* OR serialisation* OR (end-to-end NEAR/3 (verificat*)) OR (takip NEAR/3 system*) OR ((falsif*) NEAR/3 medicine* NEAR/3 directive*) OR ((radiofrequen* OR radio-frequen*) NEAR/3 identific*) OR rfid):ab, ti)

AND

(drug/exp OR ‘drug therapy’/de OR ‘drug information’/de OR ‘pharmacy (shop)’/exp OR ‘drug administration’/exp OR ‘pharmaceutics’/exp OR ‘drug industry’/exp OR ‘drug marketing’/exp OR ‘drug safety’/exp OR ‘pharmacist’/exp OR ‘prescription’/exp OR ‘drug distribution’/exp OR ‘medication error’/exp OR ‘drug labeling’/de OR ‘drug manufacture’/exp OR ‘drug monitoring’/exp OR ‘drug packaging’/exp OR ‘computerized provider order entry’/exp OR ‘medication therapy management’/de OR ‘clinical pharmacy’/de OR (drug OR drugs OR pharmaceut* OR pharmacolog* OR pharmacy* OR pharmacies* OR medication* OR pharma OR ((substandard* OR sub-standard* OR falsif*) NEAR/3 medicine*)):ab, ti)

The search yielded 2,790 articles after deduplication, which were loaded as references into EndNote. The first author read article titles, abstracts and keywords, and selected 199 candidate articles. We selected 16 articles after reading the full text.^26^ Exclusion criteria were:

1. Articles focused on one medicine specifically.
2. They expanded on a potential system rather than the actual (attempted) implementation.
3. They concerned medication or dispensing errors.
4. They described patents on (partial) PTTSs.
5. They concerned techniques or machines to print codes and labels.

**Figure 1:**
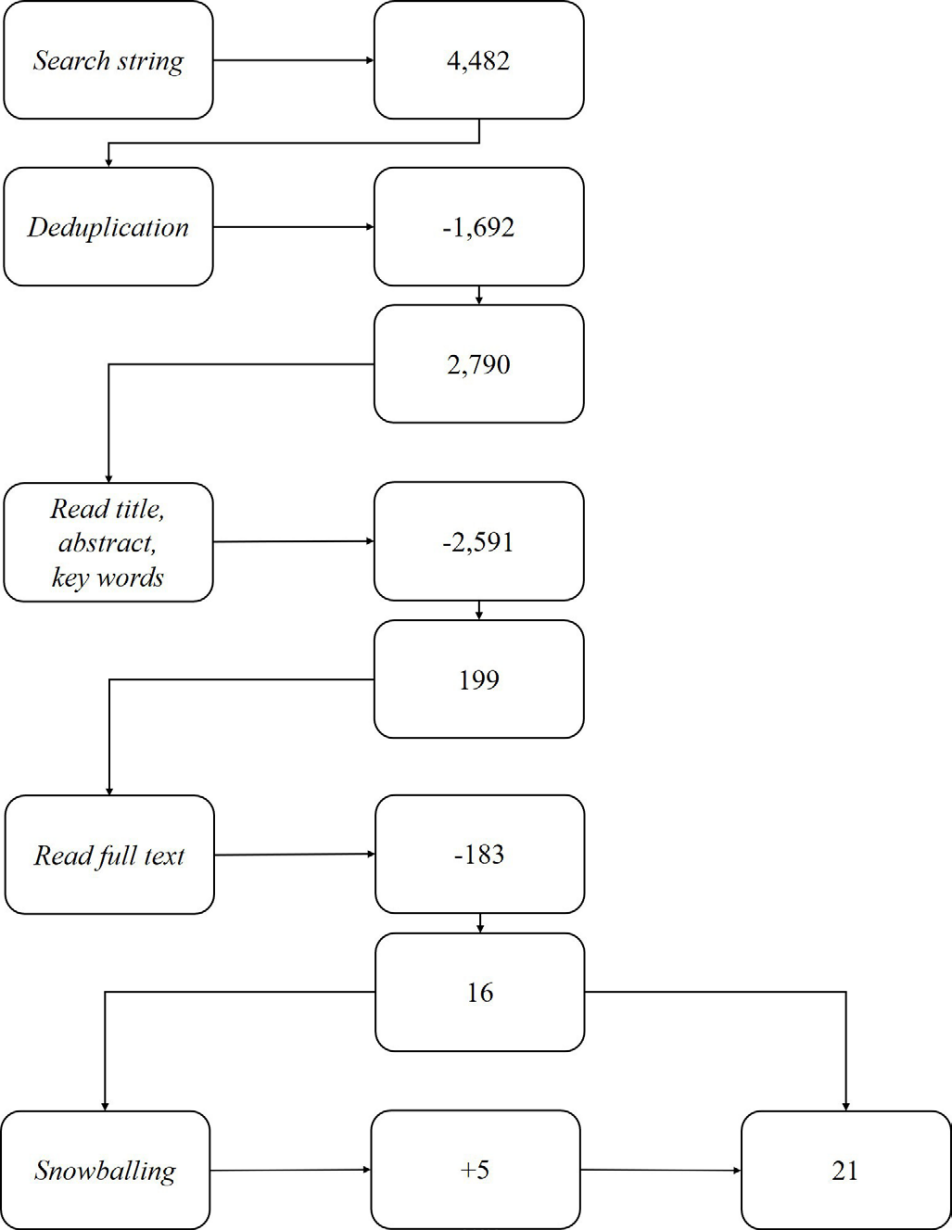
Literature search process.

The second author performed the same process for a random subset comprising 10% of the original database and resolved discrepancies through discussion with the first author. Through snowballing, 5 more articles brought the total to 21 articles (see figure 1). The first author created a Microsoft Excel table to aggregate the mechanisms and contextual factors per implemented PTTS described in the dataset.

## Results

This section will first give an overview of the implemented PTTSs described in the 21 selected articles, followed by a precis of how the articles described the contextual factors to have influenced implementation. Please note, the ‘outcome’ was equated to the success of implementation as described in the reviewed article. The focus lay on the how and why of the described interactions between contexts and mechanisms.

### Partial or full PTTSs implemented around the world

We identified six elements (‘mechanisms’) of the Turkish PTTS and compared the other systems with it. We then aggregated those elements into umbrella terms. These are: (1) a unique product code (ranging in this dataset from barcodes to data matrixes); (2) packaging requirements (coding on different layers of packaging); (3) a device for scanning the code (ranging from handheld scanners to mobile applications for smartphones); (4) a central database for storing information (ranging from national to EU-wide databases); (5) a system that cross-checks information; and (6) a warning system in case of a mismatch between purchase and sales (ranging from warning signs to disabling sales). A full PTTS mandates a unique code on all three possible levels of packaging. Primary packaging refers to an individual blister strip, secondary packaging refers to a box holding the blister strip(s), and tertiary packaging refers to a package holding several boxes of blister strips.^27^ A full PTTS also includes a scanning device and a central database that cross-checks information and communicates to the scanning device user, providing a warning in case of irregularities.^9 10^ In a full PTTS, every party handling medication (ie, manufacturers, wholesalers, pharmacies and healthcare providers) needs to verify its authenticity.^12^ The central database cross-checks the data. When a mismatch occurs, a notification goes out to the parties involved, and the sale may be aborted.^25^ Authorities may be informed when irregularities occur.^9^

Our dataset comprises articles on PTTSs implemented in 12 primarily high-income and middle-income countries, namely Denmark, Ethiopia, Germany, Hong Kong, India, Iran, Pakistan, Poland, Taiwan, Turkey, the UK and the USA. We used the article on Turkey as a benchmark. As visible in online supplemental table 1,^28^ the PTTSs varied in terms of the number of elements. The full PTTS implemented in Turkey contained all elements, but none of the other systems was equally complete; India and Ethiopia, for example, implemented pared-down versions. Several authors described unsuccessful attempts at implementing a PTTS, for example, in Pakistan and the UK.

10.1136/bmjgh-2020-003755.supp1 Supplementary data

In Turkey, our benchmark case, a full PTTS has been implemented, with a unique code on every secondary and tertiary medicine package, a scanner for every supply chain actor, and a mobile application for patients. A central database stores all information and a cross-checking system compares sales and purchasing notifications, and disables sales when notifications cannot be matched. Moreover, the Ministry of Health receives a warning about the attempted mismatch.^9^ Other countries have implemented or attempted to implement elements of a full PTTS. Denmark implemented coding for all prescription medicines, but the codes were not scanned, resulting in an incomplete database.^29^ A barcoding pilot in Ethiopia used a mobile application and a national database with real-time validation and cross-referencing, but no further implementation was realised despite promising results.^27^ A cross-country RFID-tag study was conducted in Germany, allowing all supply chain actors to communicate with a central database.^30^ In Hong Kong, case studies showed promising results for the use of RFID tags and a global sharing mechanism, including an alert in the event of unsuccessfully matched information.^31^ India implemented barcoding on drugs for export only, using codes on all packaging levels, but without a database.^32^ In Iran, the study reported pharmacists’ knowledge, practice and attitude towards RFID application.^33^ A barcode-scanning pilot was executed in Pakistan, but the results were not promising.^27^ When the paper on Poland was published, awareness was raised about the SFMP problem actively.^34^ Taiwan saw the partial and early-stage implementation of RFID tags, but no further implementation so far.^35^ In a barcode pilot in the UK, many falsified medical products remained unidentified.^36^ For the USA, we retrieved six articles, describing varying levels of legislation are implemented across states, showing various levels of success. No national, overarching system is currently in place.^37–42^ In low-income countries (LICs) in general, awareness is increasing.^43^

### Contextual factors affecting implementation

We identified six contextual factors (‘contexts’) that hampered or facilitated implementation, divided into three categories: political, economic, and social contextual factors.

Of the political contextual factors (see table 1), the first is *government support*. Our data show that government support is crucial^32^ : in Turkey, the healthcare system was facing extensive reimbursement fraud, resulting in a political sense of urgency.^9 10^ In India, the government actively initiated PTTS implementation as well.^32^ The US government was aware of the problem of SFMP in the medical supply chain but had not taken coordinated action: other stakeholders, such as pharmaceutical companies and information technology companies, initiated implementation of PTTS elements. The absence of mandated action has led to fragmented implementation across states.^37 39 40 42^ Accordingly, this category’s second factor is *legislation and regulation*, described as essential in several articles.^9 29 33 34^ Well-defined legislation and regulation eased the implementation of the PTTS in Turkey, also seen in Germany, for example, where legislation-like fines for non-compliance were imposed on the market, facilitating implementation.^30^

**Table 1:** Political contextual factors identified affecting PTTSs; no data were retrieved for Ethiopia, Hong Kong, Pakistan, Taiwan and the UK

Country	Government support	Legislation and regulation
Turkey	Governmental support and action, thanks to a political sense of urgency because of widespread reimbursement fraud.^9^	Legislation and regulation were well defined.^9^
Denmark		Legislation was becoming increasingly extensive and complex.^29^
Germany		Specific legislation and increased requirements of drug safety. Pharmaceutical companies that did not comply face fines and charges.^30^
India	Government started to realise the extent of the problem^32^…	… resulting in more extensive regulation.^32^
Iran	A general lack of concern about the topic.^33^	Little regulation was in place.^33^
Poland		Requirements mainly regarding banning the marketing of SFMP.^34^
USA	The government was aware of the SFMP problem and recommended measures,^37^ but the use of PTTS had not been mandated.^42^	A framework for the guidance of PTTS efforts had been released, but states were developing their own laws.^38^ The standards were often inconsistent among states, which may impede the adoption of PTTSs across the national supply chain.^39^

PTTS, pharmaceutical track-and-trace system; SFMP, substandard and falsified medical product.

The second category, social contextual factors (table 2), holds two elements specific to supply chain actors (like manufacturers, wholesalers and healthcare providers). The first is *supply chain actor support* for implementation.^9 29 30 32–34 37^ Several factors, like fear of change, may decrease support.^31 37^ In Turkey, mainly the costs imposed on supply chain actors made them sceptical, but (financial) incentives convinced them to comply.^9^ Support may also be related to legislation and regulation; in Denmark, the legislation’s high complexity made compliance highly demanding, decreasing support.^29 44^ In Germany, the PTTS was tailored to already existing processes, resulting in minimal changes and more significant support.^30^ Supply chain actors appear to become more supportive by training and preparation, thus gaining *awareness, knowledge, and skill*, the second social contextual factor. In Turkey, the SFMP problem was widely recognised,^9^ whereas, in both Poland and Iran, the lack of knowledge about SFMP and PTTSs contributed to a lack of action.^33 34^ Ting *et al*^45^ describe how proper training overcame the fear of change in Hong Kong.

**Table 2:** Social contextual factors identified affecting PTTSs; no data were retrieved for Ethiopia, Pakistan, Taiwan and the UK

Country	Supply chain actor support	Awareness, knowledge, and skill
Turkey	Stakeholders were sceptical at first but convinced along the way.^9^	The problem of SFMP was widely recognised.^9^
Denmark	The complexity of legislation made compliance highly demanding and set the bar too high for stakeholders.^29^	
Germany	Stakeholders were aware of upcoming requirements, but personal concerns were the main drivers for adoption.^30^	
Hong Kong	Fear of change was one of the critical barriers to implementation…^31^	… but could be overcome with decent training.^45^
India	There was no united effort from supply chain actors.^32^	
Iran		Supply chain actors did not know about PTTS or the skills required. They were not even interested in using these technologies.^33^
Poland	Supply chain actors did not support the proposed changes.^34^	There was little awareness nor consensus about the prevalence of SFMP and the upcoming European requirements.^34^
USA	Supply chain actors wanted to implement PTTS as they were concerned about the consequences for their reputation and finances when they would become involved in an SFMP crisis.^37^	There was widespread consensus about the benefits of PTTS implementations^41^ and SFMP as a severe and growing problem.^42^

PTTS, pharmaceutical track-and-trace system; SFMP, substandard and falsified medical product.

The first economic contextual factor (see table 3) is *investments* that supply chain actors must make in terms of money, time and effort. Turkey required supply chain actors to make extensive investments. However, these actors’ willingness to make them as compliance was the only way to stay in business,^43^ providing them with sufficient financial incentive. In other cases, it seems that high investments tended to hamper implementation.^31 34 36^ In Taiwan, extensive investments were imposed on supply chain actors, and the return on investment created the key barrier to implementation.^35^ In Germany, minimal investments were required, contributing to a promising study outcome.^30^ This is thus clearly linked to the first social contextual factor, supply chain actors’ support, as the financial consequence of (not) implementing a PTTS affects support. For example, in Turkey, not supporting the implementation meant losing the entire Turkish market.^9^ In Poland, supply chain actors were unsupportive as they expected to lose money.^34^ In the USA, some stakeholders were reluctant to invest as there was uncertainty on the compatibility with existing technical systems.^31 40^ The second economic factor is the *technical and digital capacities*, the lack of which often hampered implementation.^33 34 36 40^ The Turkish study did not identify this contextual factor. However, in Hong Kong, case studies with promising outcomes showed that current technical settings hampered the adoption of PTTSs.^45^ Likewise, in Pakistan, low smartphone ownership rates posed a critical barrier to PTTS implementation.^27^

**Table 3:** Economic contextual factors identified affecting PTTSs; no data were retrieved for Denmark and India

Country	Investments	Technical and digital capacities
Turkey	High costs imposed on stakeholders; however, not investing meant no sales, providing sufficient incentive to invest.^43^	
Ethiopia		One of the lowest smartphone ownership rates globally hampered adoption.^27^
Germany	Minimal investments required.^30^	Existing healthcare IT was fragmented, but this was well managed thanks to the adjustability of the system.^30^
Hong Kong	Investments were required, but the effectiveness of the investment was unclear. Implementation was costly, time-consuming and difficult.^31^	Current technical settings hampered the adoption of technologies.^45^
Iran		Current ICT structures were insufficient for the application of PTTS technologies.^33^
Pakistan		The lack of digital inventory management was a key barrier.^27^
Poland	The proposed implementation required significant investments.^34^	Pharmacies were not supported by electronic necessities.^34^
Taiwan	High initial investments were the key barrier to implementation, especially since the eventual benefits were hard to envision.^35^	
UK	Supply chain actors were expected to invest much time and workforce resources.^36^	Hospitals did not have all technical prerequisites, and hospitals in rural areas might experience problems with their internet connection.^36^
USA	Investments in terms of finances and staff necessary to implement PTTSs were extensive, and supply chain actors were hesitant of investing.^40^	Most PTTSs were not interoperable with existing computer systems used by supply chain actors.^40^

ICT, information and communications technology; IT, information technology; PTTS, pharmaceutical track-and-trace system.

## Discussion

The first part of our research question is: what political, economic, and social contextual factors facilitate or hinder the implementation of PTTSs? The literature describes several contextual factors that have hampered or facilitated a PTTS implementation. We have bracketed them as government support, supply chain actors and the coordination between them, awareness, knowledge, and skill of supply chain actors, regulation and legislation, financial investments, and technical and digital requirements. Our data show the importance of governmental support, which, together with legislation and regulation, covers the pharmaceutical regulatory capacity previously described as likely to be influential.^17^ Government support was as crucial in India, Iran and the USA as in Turkey, our benchmark case. However, Borup *et al*^15^ note that governmental support is not always straightforward when it comes to the implementation of PTTSs, denoting the importance of the exact processes of coordination. Our data also underline the role of supply chain actors. For supply chain actors to get on board, it is crucial that investments in time, money, and effort are not too high, and should not outweigh expected future revenues. This onboarding relates to and results in having awareness, knowledge, and skill, and having specific technical and digital requirements in place. In the dataset, Denmark developed legislation to implement and use PTTS, but this legislation proved so complicated that supply chain actors became reluctant to comply.^29^ In Hong Kong, supply chain actors’ attitude towards the implementation of PTTS mechanisms was positive due to proper training and preparation. However, supply chain actors still opposed implementation as the investment costs were high and benefits hard to visualise.^31^ From this, it becomes clear that not one contextual factor arose as crucial; instead, our data highlight the influence of the country-specific *interplay* between factors on implementation success.

The second part of our research question asks: what are the implications for future PTTS implementations? The consequences of this country-specific interplay are twofold, formulated as two refined programme theories. The first refined programme theory derives from the previous paragraph and holds that the incentives for all actors, both governmental and supply chain, should be aligned to facilitate implementation. Our second refined programme theory states that adjusting the PTTS to fit the context *during implementation* will facilitate the implementation process. In many of the analysed examples, the PTTS and the context were not well adjusted. The Ethiopian pilot used a smartphone application, even though Ethiopia ranked as one of the lowest in the number of smartphone users worldwide.^27^ In Hong Kong, supply chain actors needed to change their hardware applications, involving high investment costs compounded by fear of change.^31^ In Germany, customising the PTTS to the setting reduced resistance from supply chain actors and the expected investments.^30^ For the USA, Bapat and Restivo^37^ argued that incremental implementation of PTTS might help integration.^40^ We conclude that adjusting the PTTS to the context during implementation facilitates implementation.^46^ Other authors have previously described the importance of adapting interconnected technology sets to the context, in an ‘innofusion’ process.^47 48^ Specifically, our research would suggest adapting these interconnected technologies *during the implementation process*, dependent on the contextual factor interplay over time. Such nimble implementation processes may also, for example, entail offering differing incentives at different moments, combining the learnings from our two refined programme theories. These tentative conclusions on the influence of the interplay of contextual factors during PTTS implementation would benefit strongly from further research.

### Strengths and limitations

The realist review is a favourable research method for figuring out contextual factors that influence implementation,^23^ previously used in a variety of settings^49 50^ and to inform related approaches.^51^ We built on an extensive search of the peer-reviewed literature but excluded grey literature due to time constraints, which provides an avenue for further research in this relatively new research field. Moreover, the number of articles (21) selected for review shows that not many previous authors analysed the implementation of PTTSs in these terms. Naturally, this will have affected our data: our list of contextual factors is by no means exhaustive. Taking Turkey as the benchmark case will have affected our data collection by defining the mechanisms and thus the search terms. Some PTTSs described in peer-reviewed literature may have escaped our attention as a result.

Regarding the dataset, over half the papers reviewed concern high-income countries, the rest concern primarily upper and lower middle-income countries (Iran and Turkey; India and Pakistan), with one lower income country (Ethiopia). The lower middle-income and lower-income countries concerned either a pilot or a pared-down PTTS. For Ethiopia and Pakistan, all contextual factors of identified hampering introduction of a PTTS were economic factors, which might indicate primary importance of economic contextual factors for low/middle-income countries and LICs, though no such factors were present in India. Due to the sampling, the applicability of the other contextual factor types in LICs, in particular, would benefit from further research. Generally, it is likely that contextual factors will vary in strength influence per setting. This may well correlate with the country’s income status. Another notable aspect of the dataset is that it is retrospective, and technological advance happens quickly. One example of such technological advance currently underway is blockchain, which enables operations in low-trust environments.^52 53^ Consequently, the conclusions regarding, for example, the fact that government support is crucial, may become outdated in the future. Our refined programme theories, however, should hopefully not suffer likewise.

Our selected articles and analysis highlighted what technology worked under what circumstances, but our conceptualisations did not shed light on for whom it may, or may not, have worked.^23^ This is visible in how we conceptualised the stakeholders and their actions as a contextual factor: if an implementation process did not work for them, we have analysed this as a contextual factor hampering implementation. This is in line with our first original programme theory that guided our substantive and methodological choices, which holds that implemented PTTSs prevent falsified products from reaching patients. This programme theory is essentially a normative one, with implementation conceived of as a public *good*. We would recommend further qualitative research on the intricacies of PTTS implementation: not everyone may be in favour of the implementation of a PTTS,^15^ especially as we have aggregated all stakeholders bar the government under the heading ‘supply chain actors’.

## Conclusion

This realist review describes implementations of partial or full PTTSs. We have reviewed 21 articles on the (proposed) implementation of such systems in Turkey, which acted as our benchmark case, Denmark, Ethiopia, Germany, Hong Kong, India, Iran, Pakistan, Poland, Taiwan, the UK, and the USA. Specifically, we highlight the political, social, and economic contextual factors described as hindering or facilitating the (proposed) implementation in these primarily high-income and middle-income settings. The political contextual factors are first, government support; and second, legislation and regulation. The social contextual factors are first, supply chain actors support; and second, awareness, knowledge, and skill. The economic contextual factors are first, investments; and second, technical and digital requirements. Overall, we conclude that the interplay between contextual factors affects PTTS implementation strongly. Aligning incentives for all actors and leaving sufficient room for adjusting the PTTS to its context during implementation are likely to facilitate implementation.

## Data Availability

Data sharing not applicable as no datasets generated for this study: the data analysed in this study comprise previously published articles only.
