# Supplementary material for: Implementing pharmaceutical track-and-trace systems: a realist review
Source: BMJ Glob Health. 2021 May 28;6(Suppl 3):e003755. doi: 10.1136/bmjgh-2020-003755 (PMC8166635; doi:10.1136/bmjgh-2020-003755)
Supplement: Supplementary data [file bmjgh-2020-003755supp001.pdf]

Supplementary Table 1 – Reviewed PTTS implementations. Letters a-t refer to study reviewed (please see below for the complete list). Numbers 1-6 in the first column indicate elements of a PTTS (derived from [u]). Countries are sorted based on the income level of countries according to the United Nations Development Program (http://hdr.undp.org/en/content/income-index), with the four highest in table 1a, the middle four in table 1b, and the lowest in table 1c.

| **Supp. table 1a** | USA | Germany | Denmark | UK |
|---|---|---|---|---|
| Implementation status | Requirements implemented in 29 states, 6 states have pending legislation [a]; Implementation varies between states and hospitals [b,c]. | Cross- country study, not yet nationally implemented [h]. | Coding is implemented [i]; using the codes is not implemented [j]. | Pilot in a hospital [k]. |
| Level of success | The pilots and partial implementation show various levels of success, but the fact that no overarching, national system is in place does not indicate great success. | Promising results, implementation seems beneficial [h]. | The current system is functioning, but not up to EU standards [i,j]. | No promising results; many SFMP remained unidentified [k]. |
| 1. A unique code | RFID is the most frequently mentioned method of serialisation [a-f]. | RFID-tags [h]. | Barcodes on all prescription medicines [j]. | 2D barcodes and authentication logo for pharmacies [k]. |
| 2. Packaging requirements | | Attached to “the products” [h]. | On “each package of medicine” [j]. | |
| 3. A scanning device | RFID- readers [d,f]. | RFID readers in the form of a specialised terminal, also able to perform bulk readings [h]. | Scanning is not yet implemented [j]; data is inserted manually [i]. | Scanning [k]. |
| 4. A central database | No national, overarching system, but in some pilots, databases used to store information and allows correspondence [c,g]. | Central database ‘The Pharmaceutical Trust Center’ [h]. | In the future: an EU-wide database [j]; now there is a national database [i]. | |
| 5. A cross-checking system | | Medical product can be shipped when products are serialised and stored in the database [h]. | | |
| 6. A warning/ disabling function | In one pilot, discrepancies are reported to the distributor [g] | | | |

| **Supp. table 1b** | Hong Kong | Poland | Turkey | Iran |
|---|---|---|---|---|
| Implementation status | Case studies, no national implementation [l,m]. | Nothing implemented, but awareness is raised [n]. | Implemented throughout the supply chain [o-q]. | Research, nothing implemented [r]. |
| Level of success | Promising results but no further implementation [l,m]. | | Very successful [o-q]. | |
| 1. A unique code | RFID-tags [l,m]. | | 2D datamatrix [o,q]. | |
| 2. Packaging requirements | Tag inside “each product package” [l]. | | A code on every secondary and tertiary packaging [o]. | |
| 3. A scanning device | Scanning with RFID reader [l]. | | Datamatrix scanner for supply chain actors and mobile application for patients [o]. | |
| 4. A central database | Global sharing mechanism [l,m]. | | Central database [o]. | |
| 5. A cross-checking system | Messages between supply chain parties must match [l]. | | Sales- and purchasing notifications are compared to cross-check movements [o]. | |
| 6. A warning/ disabling function | An alert is provided in the event of unsuccessfully matched information [l]. | | Completing a sale is not possible when the notifications do not match and a warning is sent to the Ministry of Health [o]. | |

| **Supp. table 1c** | Taiwan | India | Pakistan | Ethiopia |
|---|---|---|---|---|
| Implementation status | Partial implementation: in limited number of hospital and in early adoption stage [s]. | Only implemented for exported drugs [t]; implementation attempts [a]. | Pilot [u]. | Pilot [u]. |
| Level of success | Hesitation for further implementation [s]. | | No promising results as data were not captured properly [u]. | Promising results, but no implementation [u]. |
| 1. A unique code | RFID-tags [s]. | 1D or 2D barcodes [a]. | 2D barcodes [u]. | Barcodes [u]. |
| 2. Packaging requirements | | Code on primary, secondary and tertiary packaging levels [a]. | Code on secondary packages [u]. | Code on secondary packages [u]. |
| 3. A scanning device | RFID reader with software application [s]. | | Handheld scanners [u]. | Mobile application using telephone camera [u]. |
| 4. A central database | Back-end application that stores information [s]. | No database [t]. | | National database [u]. |
| 5. A cross-checking system | | | Web- interface with global information [u]. | Real-time validation and cross-referencing [u]. |
| 6. A warning/ disabling function | | | | |

References:


a. Bansal, D., et al., Anti-Counterfeit Technologies: A Pharmaceutical Industry Perspective. Scieåntia Pharmaceutica, 2013. 81(1): p. 1-13.
b. Ducca, A.T., Prescription drug counterfeiting: distributors meet the challenge. Journal of Pharmacy Practice, 2006. 19(4): p. 230-235.
c. Fisher, J.A. and T. Monahan, Tracking the social dimensions of RFID systems in hospitals. International journal of medical informatics, 2008. 77(3): p. 176-183.
d. Bapat, V. and G. Restivo, Reaping the long-term benefits of integrating radio frequency identification (RFID) into pharmaceutical manufacturing. Pharmaceutical Engineering, 2005.
e. Bernstein, I.B. and J. Shuren, The Food and Drug Administration's Counterfeit Drug initiative. Journal of Pharmacy Practice, 2006. 19(4): p. 250-254.
f. Wyld, DC and M.A. Jones, RFID is no fake: the adoption of radio frequency identification technology in the pharmaceutical supply chain. International Journal of Integrated Supply Management, 2007. 3(2): p. 156-171.
g. Jayaraman, R., et al., An Exploratory Pilot Study on Supply Chain Data Standards in a Hospital Pharmacy. Engineering Management Journal, 2015. 27(3): p. 141-151.
h. Chircu, A., E. Sultanow, and SP Saraswat, Healthcare RFID in Germany: an integrated pharmaceutical supply chain perspective. Journal of Applied Business Research (JABR), 2014. 30(3): p. 737-752.
i. Wallach Kildemoes, H., H. Toft Sørensen, and J. Hallas, The Danish national prescription registry. Scandinavian journal of public health, 2011. 39(7_suppl): p. 38-41.
j. Borup, R., et al., Fighting falsified medicines with paperwork–a historic review of Danish legislation governing distribution of medicines. Journal of pharmaceutical policy and practice, 2016. 9(1): p. 30.
k. Frontini, R., Falsified Medicines Directive: are we heading in the right direction? 2017, SAGE Publications Sage UK: London, England.
l. Kwok, S., et al., A counterfeit network analyser based on RFID and EPC. Industrial Management & Data Systems, 2010.
m. Ting, S., et al., Critical elements and lessons learnt from the implementation of an RFID-enabled healthcare management system in a medical organisation. Journal of medical systems, 2011. 35(4): p. 657-669.
n. Merks, P., et al., The European Falsified Medicines Directive in Poland: background, implementation and potential recommendations for pharmacists. European Journal of Hospital Pharmacy, 2016. 25(1): p. 10-15.
o. Parmaksiz, K., Pisani, E., & Kok, M. O. (2020). What makes a national pharmaceutical track and trace system succeed? lessons from turkey. Global Health: Science and Practice, 8(3), 431-441.
p. Pisani, E., Nistor, A. L., Hasnida, A., Parmaksiz, K., Xu, J., & Kok, M. O. (2019). Identifying market risk for substandard and falsified medicines: an analytic framework based on qualitative research in China, Indonesia, Turkey and Romania. Wellcome open research, 4.Tekiner H. Pharmacy in Turkey: past, present, and future. Pharmazie, 2014. 69(6): p. 477-80.
q. Bastani, P., et al., The Pharmacists' Knowledge, Attitude and Practice Toward the Application of Radio-frequency Identification in Iranian Pharmacies. International Journal of Pharmaceutical Investigation, 2018. 8(1): p. 44-49.
r. Lai, H.-M., I.-C. Lin, and L.-T. Tseng, High-level managers' considerations for RFID adoption in hospitals: an empirical study in Taiwan. Journal of medical systems, 2014. 38(2): p. 3.
s. Moniveena, M.G., Pramod Kumar, T. M., An overview of track & trace regulations in pharma industry and its impact on the reverse logistics of medicines- status in regulated countries and India. International Journal of Pharmaceutical Sciences Review and Research, 2017. 47(2): p. 85-91.
t. Hara, L., et al., *More Than Bar Codes: Integrating Global Standards-Based Bar Code Technology Into National Health Information Systems in Ethiopia and Pakistan to Increase End-to-End Supply Chain Visibility.* Global Health: Science and Practice, 2017. **5**(4): p. 678-685.
u. Kootstra, J.G., The implementation of a pharmaceutical track-and-trace system in a new context: Matching inside out. 2019, Erasmus Universiteit Rotterdam.
